# Photosynthetic Responses of Freshwater Macrophytes to the Daily Light Cycle in Songkhla Lagoon

**DOI:** 10.3390/plants11212806

**Published:** 2022-10-22

**Authors:** Ponlachart Chotikarn, Pathompong Pramneechote, Sutinee Sinutok

**Affiliations:** 1 Marine and Coastal Resources Institute, Faculty of Environmental Management, Prince of Songkla University, Songkhla 90110, Thailand; 2 Faculty of Environmental Management, Prince of Songkla University, Songkhla 90110, Thailand; 3 Coastal Oceanography and Climate Change Research Center, Prince of Songkla University, Songkhla 90110, Thailand

**Keywords:** macrophytes, photosynthesis, rapid light curves, Songkhla Lagoon

## Abstract

Macrophytes play an important role in the freshwater ecosystem. However, human activities and climate change are currently affecting aquatic lakes and species in various ways. The heterogeneity of macrophyte ecophysiology might lead to different responses to changing environments. To understand the photosynthetic responses of freshwater macrophytes to changes in light, six freshwater macrophyte species from Songkhla Lagoon were investigated. The results showed that there was a diurnal response of photosynthetic activities in all species. The F_v_/F_m_ and ∆F/F_m_′__ of all species decreased at midday (9 a.m.–3 p.m.) then recovered at 6 p.m. close to the 6 a.m. level. As well as alpha, the positive relation between alpha and light showed the adaptation of plants. The saturating irradiance (I_k_) revealed that plants from different light regimes showed different responses to light and temperature changes. To maintain a positive carbon balance and cope with light and temperature conditions, macrophytes had strategies such as modifying light harvesting capacity and light use. This study provides a better understanding of the vulnerability of each species to environmental changes and photosynthetic responses that enable species from different light regime to adapt to changing light environments.

## 1. Introduction

Macrophytes play an important role in the function of freshwater ecosystems by maintaining biological diversity, acting as ecosystem engineers [1]; habitat structure for other aquatic organisms [2]; and affecting lake nutrient status, sediment resuspension, and water turbidity [3,4]. However, human activities and climate change are currently affecting aquatic lakes and species through several factors, such as eutrophication, habitat destruction, and changes in water chemistry and physical properties [5].

Thale Noi is one of four interconnected water bodies in the Songkhla Lagoon, which is a tropical estuarine lagoon system covering 27 km^2^. Thale Noi, listed as a Ramsar site, is a valuable freshwater habitat supporting not only biodiversity, but also local inhabitants whose livelihoods depend on that biodiversity via fishery, aquaculture, and tourism [6,7,8]. As Thale Noi receives runoff and wastewater including sediments from the surrounding watershed, they are currently facing serious water pollution and eutrophication problems, which may lead to losing their valuable ecosystem services and function [6,7]. Eighteen dominant species of macrophytes have been listed in Songkhla Lagoon, including floating plants *Eichhornia crassipes* and *Salvinia cucullata*; plants with roots with floating leaves *Nymphaea lotus* and *Nymphaea stellata;* and submerged plants *Ceratophyllum demersum* and *Chara zeylanica* [8,9,10]. These systems are dynamic because of their complex physical and chemical processes in water bodies and sediment. Variations in environmental factors affect aquatic macrophytes in many ways, including distribution and abundance [11]. Some macrophytes can grow very well, which leads to overgrowth that might disturb the ecosystem. Several environmental parameters reveal the dynamics in terms of diurnal such as light and temperature, which affect the growth and photosynthetic activities of macrophytes [12,13].

Rapid light curves (RLCs) are a useful tool for evaluating a plant’s photosynthetic response to varying light conditions based on the background light to which they have been acclimated [14,15]. Jiang et al. [16] found that at midday under ambient light, the maximum quantum yield (F_v_/F_m_) of six submerged macrophytes decreased, but did not significant change under shade; F_v_/F_m_ was negatively correlated with photon radiance. Shading decreased the net photosynthesis in *C. aspera* and *C. canescens* within 24 h; however, their photosynthetic performance was recovered within a short period [17]. Chen et al. [12] revealed that *Potamogeton maackianus* and *Vallisneria natans* decreased their minimum saturating irradiance (E_k_) and maximum relative electron transport rate (rETR_max_) and increased their initial slope (α) of RLCs under low light stress.

The heterogeneity of macrophyte morphology (e.g., emerged, floating, roots with floating leaves, and submerged plants) is related to water velocity and light regime [18,19]. Light and temperature are two of the most important factors controlling the growth, productivity, abundance, distribution, and survival of different groups of aquatic macrophytes [20,21,22]. The ecophysiological response of macrophytes to light and temperature depends upon their life form, traits, photosynthetic structure, and mechanisms, e.g., pigment contents, C_3_ and C_4_ plants, and their acclimation capacity to changing light and temperature [23,24,25]. The loading of sediment and nutrients and epiphytic algae can also reduce light availability and inhibit physiological processes [22]. Emergent and floating-leaved, and low-light-tolerant submerged species are commonly found in turbid water, where the presence of other submerged species is limited [3]. The temperature and minimum light requirements of a species can also determine the macrophyte depth zonation [26,27].

Understanding the photosynthetic responses of freshwater macrophytes to changes in light may explain the dynamics of these ecosystems. In this study, we investigated the photosynthetic responses of six freshwater macrophyte species from different light regimes (*Eichhornia crassipes*, *Salvinia cucullata*, *Nymphaea lotus*, *Nymphaea stellata*, *Ceratophyllum demersum,* and *Chara zeylanica*) from Thale Noi, Songkhla Lagoon. This study focused on the photosynthetic strategies that enable species to adapt to changing light environments.

## 2. Results

### 2.1. Physical Parameters

**Figure 1:**
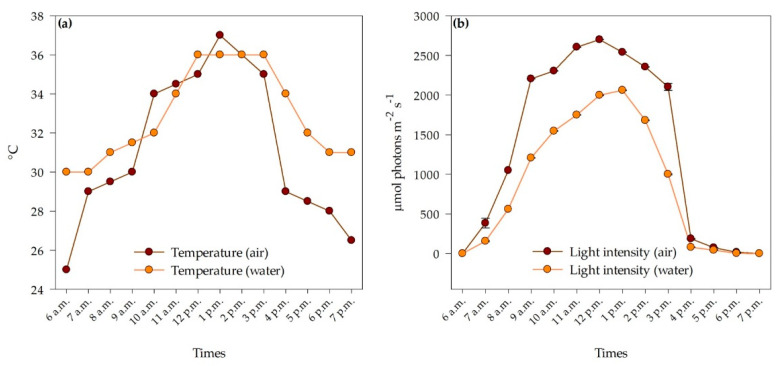
Air and water temperatures (**a**) and light intensity of air and water (**b**) at Thale Noi, Songkhla Lake from 6 a.m. to 7 p.m. (*n* = 3). Data represent mean ± SE.

The average air and surface water temperatures were 31.21 ± 1.04 and 32.89 °C ± 0.63 °C, respectively. The average light intensity of surface water and air were 863.52 ± 221.02 and 1323.32 ± 309.63 µmol photons m^−2^ s^−1^, respectively. The highest surface water and air temperatures were 36 °C at 12 – 3 p.m. and 37 °C at 1 p.m., respectively. The light intensity of the surface water and air was highest at 1 p.m. (2060.67 µmol photons m^−2^ s^−1^) and 12 p.m. (2700 µmol photons m^−2^ s^−1^), respectively (Figure 1).

### 2.2. Maximum Quantum Yield (F_v_/F_m_)

**Figure 2:**
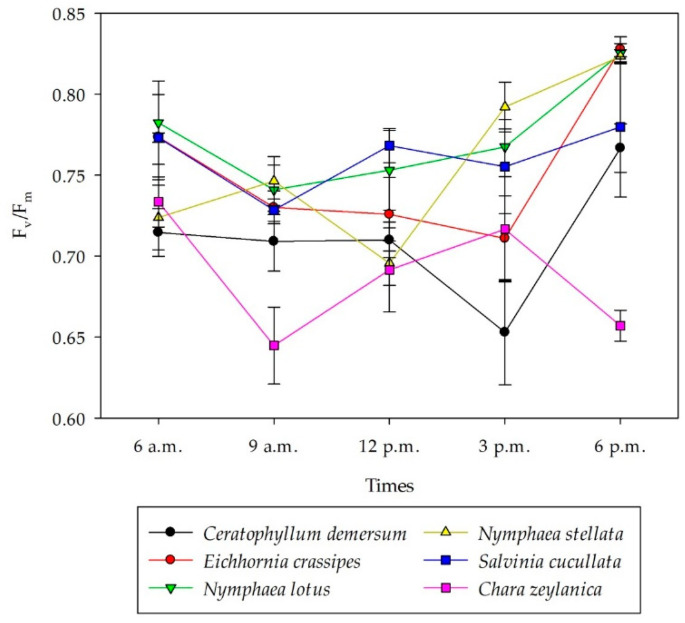
F_v_/F_m_ of six macrophyte species at Thale Noi, Songkhla Lake, from 6 a.m. to 6 p.m. for each species (*n* = 4). Data represent mean ± SE.

At 6 a.m., F_v_/F_m_ of *C*. *demersum*, *N*. *stellata*, *C*. *zeylanica, S*. *cucullata*, *E*. *crassipes*, and *N*. *lotus* were 0.71 ± 0.01, 0.72 ± 0.02, 0.73 ± 0.02, 0.77 ± 0.00, 0.77 ± 0.03, and 0.78 ± 0.03, respectively (Figure 2). A significant difference of F_v_/F_m_ among species and time were observed (*p* < 0.05) (Table 1) where F_v_/F_m_ of submerged plants (*C*. *demersum* and *C*. *zeylanica*) was significantly lower than the floating plants (*Eichhornia crassipes, Salvinia cucullata*) and root with floating leaf plant (*Nymphaea lotus*, *Nymphaea stellata*) (*p* < 0.05). Results showed that there was no difference between 6 a.m. and 6 p.m. and 9 a.m. and 3 p.m. (*p* > 0.05), revealing diurnal recovery and down-regulation in midday, respectively. A comparison among species found that roots with floating leaves (*N*. *lotus* and *N*. *stellata*) and floating plants (*E. crassipes* and *S. cucullata*) had a decreased tendency in F_v_/F_m_ at midday, while submerged plants (*C*. *demersum* and *C*. *zeylanica*) showed various trends.

### 2.3. Rapid Light Curves (RLCs)

**Figure 3:**
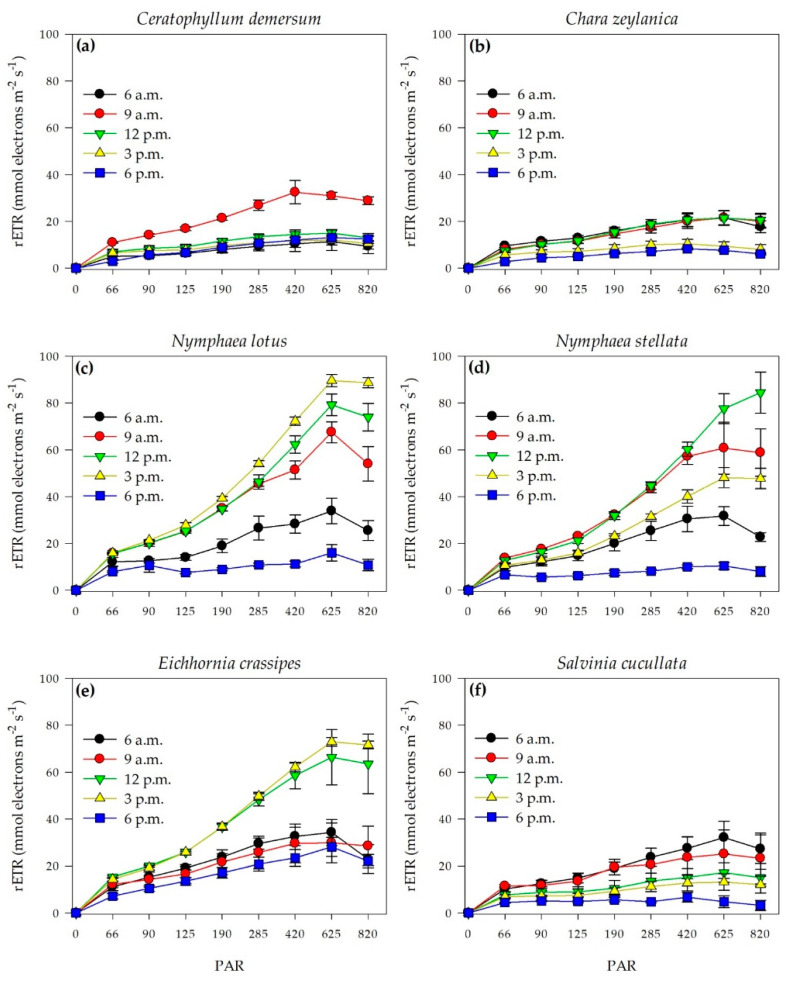
RLCs of six macrophyte species at Thale Noi, Songkhla Lake, from 6 a.m. to 6 p.m. for *Ceratophyllum demersum* (**a**), *Chara zeylanica* (**b**), *Nymphaea lotus* (**c**), *Nymphaea stellata* (**d**), *Eichhornia crassipes* (**e**), and *Salvinia cucullata* (**f**) (*n* = 4). Data represent mean ± SE.

The RLCs showed responses to the light regime and revealed downregulation of photosynthesis for each submerged plant. *C*. *demersum* had the highest relative electron transport rate (rETR) at 9 a.m., while *N. lotus* and *N*. *stellata* had the highest at 3 and 12 p.m., respectively. The rETR of *C*. *zeylanica*, *S*. *cucullata*, and *E*. *crassipes* was high in the range of 6 a.m.–12 p.m., 6–9 a.m., and 12–3 p.m., respectively (Figure 3). However, all species had a low rETR at 6 p.m.

#### 2.3.1. Effective Quantum Yield (∆F/F_m__′_)

**Figure 4:**
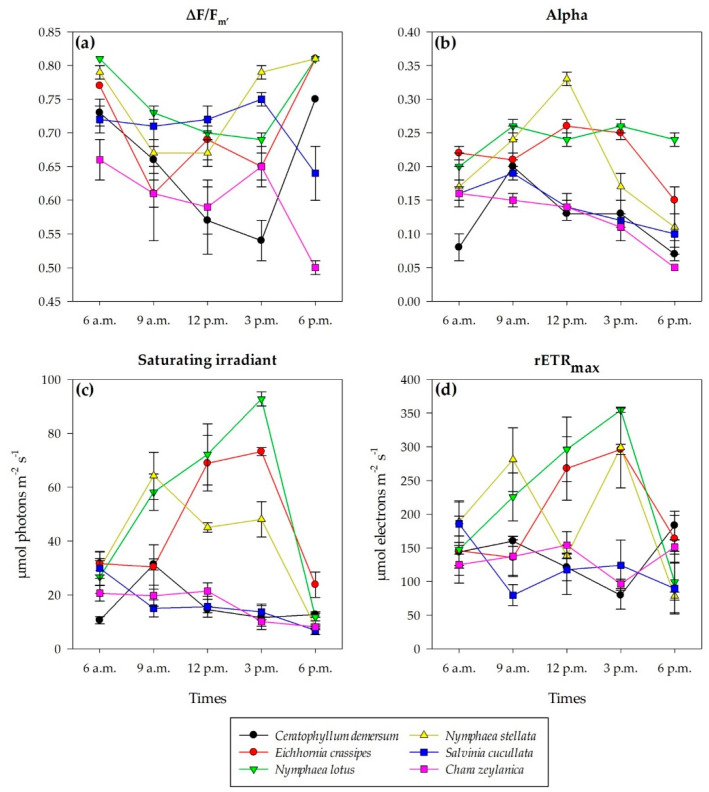
∆F/F_m__′_ (**a**), alpha (**b**), I_k_ (**c**), and rETR_max_ (**d**) of six macrophyte species at Thale Noi, Songkhla Lake, from 6 a.m. to 6 p.m. for each species (*n* = 4). Data represent mean ± SE.

There were significant changes in the ∆F/F_m__′_ in all species (*p* < 0.05) except for *S*. *cucullata* during the day (*p* > 0.05). At 6 a.m., the ∆F/F_m__′_ of *C*. *zeylanica*, *S*. *cucullata*, *C*. *demersum*, *E*. *crassipes*, *N*. *stellata*, and *N*. *lotus* were 0.66 ± 0.03, 0.72 ± 0.02, 0.73 ± 0.02, 0.77 ± 0.00, 0.79 ± 0.01, and 0.81 ± 0.00, respectively (Figure 4a). Then, the ∆F/F_m__′_ of *C*. *demersum*, *E*. *crassipes*, and *N*. *lotus* significantly decreased from 6 a.m. to 3 p.m. and significantly increased at 6 p.m., while the ∆F/F_m__′_ of *N*. *stellata* and *C*. *zeylanica* significantly increased at 3 p.m. The ∆F/F_m__′_ was different among species and time (*p* < 0.05) (Table 1), where the ∆F/F_m__′_ of submerged plants (*C*. *demersum* and *C*. *zeylanica*) was significantly lower than that of the other species (*p* < 0.05). In all species except *C*. *zeylanica*, the ∆F/F_m__′_ at 6 a.m. and 6 p.m. was not significantly different, indicating the recovery of photosynthetic efficiency.

#### 2.3.2. Alpha (α)

At 6 a.m., the α values of *C*. *demersum*, *C*. *zeylanica*, *S. cucullata*, *N*. *stellata*, *N*. *lotus*, and *E*. *crassipes* were 0.08 ± 0.02, 0.16 ± 0.00, 0.16 ± 0.01, 0.17 ± 0.03, 0.20 ± 0.02, and 0.22 ± 0.01, respectively (Figure 4b). The α in all species significantly decreased over time at midday (*p* < 0.05), except in *N*. *lotus* (*p* > 0.05), which showed a slightly stable trend. The α was different among species and time (*p* < 0.05) (Table 1), where *N*. *lotus* and *N*. *stellata* showed the slowest change in α, while the fastest change occurred in submerged plants (*C*. *demersum* and *C*. *zeylanica*). There was no difference in the α between 6 a.m. and 6 p.m. (*p* > 0.05), revealing that all plants could adapt to cope with ambient light and return to their normal state.

#### 2.3.3. Saturating Irradiance (I_k_)

At 6 a.m., the saturating irradiance (I_k_) of *C*. *zeylanica*, *C*. *demersum*, *E*. *crassipes*, *N*. *lotus*, *S. cucullata* and *N*. *stellata* was 125.04 ± 15.76, 143.81 ± 24.01, 145.71 ± 21.85, 147.45 ± 49.82, 185.60 ± 32.21, and 188.69 ± 30.93 μmol photons m^−2^ s^−1^, respectively (Figure 4c). There were significant changes in the I_k_ in all species (*p* < 0.05), except for *S. cucullata* and *C*. *zeylanica* (*p* > 0.05). The I_k_ of submerged plants (*C*. *demersum* and *C*. *zeylanica*) decreased at noon, the I_k_ of *E*. *crassipes* and *S. cucullata* decreased at 9 a.m. and 6 p.m., and the I_k_ of *N*. *lotus* and *N*. *stellata* decreased only at 6 p.m. The I_k_ significantly differed among species and time (*p* < 0.05) (Table 1). The I_k_ at 6 p.m. was not significantly different from the I_k_ at 6 and 9 a.m., indicating the recovery of photosynthetic efficiency.

#### 2.3.4. Maximum Relative Electron Transport Rates (rETR_max_)

At 6 a.m., the rETR_max_ of *C*. *demersum*, *C*. *zeylanica*, *N*. *lotus*, *N*. *stellata*, *S*. *cucullata*, *E*. *crassipes* was 10.62 ± 1.37, 20.62 ± 2.90, 26.66 ± 6.06, 29.84 ± 3.70, 29.88 ± 6.25, and 31.66 ± 4.28 µmol electrons m^−2^ s^−1^, respectively (Figure 4d). There were significant changes in the rETR_max_ over time in all species (*p* < 0.05). The rETR_max_ showed various patterns for each species, in which *C*. *demersum* and *N*. *stellata* had the highest rETR_max_ at 9 a.m., those of *E*. *crassipes* and *N*. *lotus* were highest at 3 p.m., and those of *S*. *cucullata* and *C*. *zeylanica* were the highest at 6 a.m.–12 p.m., which then decreased after the peak (Figure 4d). The rETR_max_ was significantly different among species and time (*p* < 0.05) (Table 1).

## 3. Discussion

Photosynthetic responses to the daily light cycle in six freshwater macrophyte species, *Eichhornia crassipes*, *Salvinia cucullata*, *Nymphaea lotus, Nymphaea stellata*, *Ceratophyllum demersum,* and *Chara zeylanica* at Thale Noi, Songkhla Lake, were investigated. We observed a diurnal response of photosynthetic activities in all species.

The maximum quantum yield (F_v_/F_m_) and effective quantum yield (∆F/F_m__′_) indicate the diurnal change in which photosynthetic efficiency decreases during midday and returns to the morning level at the end of the day due to photoinhibition, photoprotection, and photoadaptation [16,27,28]. Diurnal changes in F_v_/F_m_ were also observed in *Lagarosiphon major*, indicating the dynamic photoinhibition of PSII [27]. Excessive photosynthetically active radiation (PAR) and a high air temperature during midday might lead to the downregulation of photosynthesis and a change in the balance of energy absorption and dissipation of PSII, resulting in photoprotection against photodamage [16]. This is consistent with the results of the study conducted by Chotikarn et al. [29], reporting that an elevated temperature of 36 °C resulted in the downregulation of photosynthesis in *C. demersum*. Such high air and water temperatures might degrade photosystem II in the photosynthetic apparatus [29]. Furthermore, the recovery of F_v_/F_m_ at 6 p.m. indicated the reversible photoinhibition, effective photoprotection, and photoadaptation of PSII of these macrophyte species. The ability of aquatic plants to survive under various conditions of light availability is partly related to their form, traits, photosynthetic structure, and mechanisms such as pigment contents, C_3_ and C_4_, and acclimation capacity to changing light and temperature [11,23,24,25,30]. These species contain chlorophyll *a* and *b* as light harvesting pigments and carotenoid as a photoprotection mechanism. Prior studies reported that the chlorophyll *a* to chlorophyll *b* ratio and chlorophyll *a* to carotenoid ratio indicate the size of light harvesting complexes and photoprotection capacity [31] and vary according to light and temperature changes. Changes in the pigment contents and these ratios may be further analyzed to confirm the photosynthetic strategy of these macrophytes.

The saturating irradiance (I_k_) reflects the minimum light that saturates a plants’ capacity for photosynthesis. The results showed that the responses of plants located in the same position in the water exposed to a similar light regime were similar; for example, floating plants and roots with floating leaves that were light-adapted showed an increased I_k_ when light intensity was high. In contrast, the I_k_ of the submerged plants that were shade-adapted had a negative relationship with light intensity, and the I_k_ increased again at 6 p.m. due to the plants’ acclimation to prevent photodamage by excessive light. There is a different light intensity between under and above water surfaces, and underwater light was in the range of 40–80% of the ambient surface light in this study. This resulted in plants in different positions experiencing different light regimes. Lacoul and Freedman [11] reviewed that some species of submerged macrophytes are highly intolerant to shading, such as *Chara* spp. and *Potamogeton alpinus*, while others may grow in gaps beneath a well-established canopy, such as *Ceratophyllum demersum*, *Hydrilla verticillata*, *Myriophyllum spicatum*, *Potamogeton crispus*, *P. pectinatus*, and *Urtricularia vulgaris* [32,33]. 

To maintain a positive carbon balance and cope with light conditions, macrophytes have developed strategies such as modifying light harvesting capacity, light use efficiency, and rates of growth [34]. For example, when light is limited, plants might increase their chlorophyll content to enhance photosynthesis efficiency. In contrast, chlorophyll content may be decreased due to photodamage under high light conditions. For *S*. *cucullata*, there was no change in the I_k_ during the daily light cycle. However, there was an adjustment in photophysiology. The decrease in alpha (α) revealed that *S*. *cucullata* has a wide range of light tolerance. 

The ∆F/F_m__′_ differed between species, which might indicate differences in light harvesting efficiency. In this study, the change in alpha revealed plants’ adaptation related to light use efficiency and flexibility to cope with different light regimes. There was a change in alpha for all species except *N*. *lotus,* revealing that *N*. *lotus* might be a wide-range light-tolerant plant whose photosynthesis efficiency is high even in full sunlight at 12 p.m. *N. lotus* exhibits sun plant characteristic and is able to maintain high rates of photosynthesis in full sunlight [35]. A similar study was conducted by Ritchie [35], who found that the F_v_/F_m_, rETR_max_, and quantum efficiency of *Nymphaea caerulea* Saligny all vary on a diurnal cycle. The maximum relative electron transport rate (rETR_max_) showed various responses, which depended on each species [35]. The highest rETR_max_ for each species in this study occurred at different times and, in all species, was related to ∆F/F_m__′_. After the rETR_max_ reached the peak, the ∆F/F_m__′_ of *E*. *crassipes,* and *S*. *cucullata* decreased at 3 p.m. and 6 a.m., respectively; those of *N*. *lotus* and *N*. *stellata* decreased at 3 p.m. and 9 a.m., respectively; and those of *C*. *demersum* and *C*. *zeylanica* decreased at 9 a.m. and 12 p.m., respectively. This relationship revealed a downregulation of photosynthesis, which decreased the light harvesting efficiency of plants [16].

Comparing the species, strategy for each species, and diurnal photosynthesis response, we found that *E. crassipes*, with their light stress tolerance and rapid growth rate, was a competing plant in the lake and tend to dominate in Songkhla Lagoon. This free-floating plant can monopolize light and absorb nutrients from the water column [36]. The extensive growth of *E*. *crassipes* has negative impacts on socioeconomic perspectives such as boating access, navigability and recreation, access to water supply for agriculture, and disturbed breeding or feeding in some living organisms. Thus, Songkhla Lagoon management should focus on controlling *E*. *crassipes*. Various applications of *E*. *crassipes* as biomass and biodiesel [37,38], electrode material [39,40], and heavy metal remover [41] have been investigated. However, high light exposure for submerged macrophytes should be avoided during lake restoration to prevent photoinhibition and photodamage. This study provides a better understanding of the photosynthetic response that enables species to adapt to changing light environments.

## 4. Materials and Methods

### 4.1. Study Site

Study site was located in Thale Noi (7^º^47′06″ N and 100^º^8′9.999″ E), Songkhla Lagoon, Thailand. Photosynthetic responses of six freshwater macrophyte species (*Eichhornia crassipes, Salvinia cucullata*, *Nymphaea lotus*, *Nymphaea stellata*, *Ceratophyllum demersum*, and *Chara zeylanica)* were investigated in April 2017. All six macrophyte species were collected (*n* = 4) from the same area, so interspecies comparisons of photobiology were therefore possible. Samples were maintained in freshwater during measurements. The measurement of maximum quantum yield of PSII (F_v_/F_m_) was made on the plant leaves after dark adaptation, and the rapid light curves (RLCs) measurements were made within 5 min of collection. Air and surface water temperature and irradiance were recorded hourly from 6 a.m. to 7 p.m. using a YSI Pro Plus multiparameter (YSI Incorporated, Ohio, USA) and Light Sensor Logger (LI-1500, LI-COR Environment, Nebraska, USA) (*n* = 3).

### 4.2. Maximum Quantum Yield (F_v_/F_m_)

Photosynthetic activity, as maximum quantum yield (F_v_/F_m_), was recorded by estimating chlorophyll a fluorescence using a Pulse Amplitude Modulated (MINI-PAM) fluorometer (Walz, Germany). Dark-adapted photosystem II (PSII) photochemical efficiency was measured as maximum quantum yield (F_v_/F_m_) after using a dark-adapted chamber for 15 min (*n* = 4). F_v_/F_m_ was calculated from (F_m_ − F_0_)/F_m_, where F_0_ represents the minimum fluorescence of dark-adapted biological materials, and F_m_ represents the maximum fluorescence after a saturating pulse is applied [42]

### 4.3. Rapid Light Curves (RLCs)

The photosynthetic performance of all six species was examined by performing RLCs using a PAM fluorometer (MIMI-PAM, Walz, Germany) (*n* = 4). RLCs with 9 increasing actinic light intensities (0, 66, 90, 125, 190, 285, 420, 625, and 820 μmol photons m^−^^2^ s^−^^1^) were applied, with 0.8 s saturating flashes (>4500 μmol photons m^−^^2^ s^−^^1^) between each actinic light intensity every 10 s [43]. The effective quantum yield of PSII (ΔF/F_m__′_ was calculated from (F_m__′_ – F)/ F_m__′_, where F represents the minimum fluorescence of light-adapted biological materials, and F_m__′_ represents the maximum fluorescence after a saturating pulse is applied [44]). The maximum relative electron transport rate (rETR_max_) was calculated using ΔF/F_m__′_ × photosynthetically active radiation (PAR, measured in μmol photon m^−2^ s^−1^). The minimum saturating irradiance (I_k_) and initial slope (α) of the RLCs were calculated using the curve fitting protocols outlined by Ralph and Gademann [14] and Sinutok et al. [43].

### 4.4. Data Analysis

Repeated ANOVA tests were performed to determine any significant differences in chlorophyll fluorescence parameters (ΔF/F_m__′_, rETR_max_, I_k_, α) among times and species, with a significant level of 95%. If data did not meet the assumptions of normality (Kolmogorov–Smirnov test) and equal variance (Levene’s test), the data were transformed using log^10^ or square root.

## 5. Conclusions

The photosynthetic responses of six freshwater macrophyte species were investigated. There was a significant difference among species and times, which revealed diurnal response and adaptation. The F_v_/F_m_ and ∆F/F_m__′_ of all species decreased at midday (9 a.m.–3 p.m.), then returned to 6 a.m. levels by 6 p.m. Our results revealed that submerged plants and water surface plants showed different responses to cope with the light regimes. Changing light intensity and temperature in the future due to climate and anthropogenic changes might affect species composition and zonation in lakes. Further research on biochemical and physiological stress responses, photoprotective pigments, and enzyme activity for each species is thus warranted.

## Figures and Tables

**Table 1:** Two-way repeated ANOVA summary table.

Parameters	Interaction	SS	df	MS	F	*p*
F_v_/F_m_	Species	0.236	5	0.047	36.714	<0.001*
	Error (Species)	0.023	18	0.001		
	Time	0.060	4	0.015	14.619	<0.001*
	Species * Time	0.094	20	0.005	4.568	<0.001*
	Error (Time)	0.074	72	0.001		
∆F/F_m__′_	Species	0.326	5	0.065	26.376	<0.001*
	Error (Species)	0.045	18	0.002		
	Time	0.143	4	0.036	14.572	<0.001*
	Species * Time	0.335	20	0.017	6.827	<0.001*
	Error (Time)	0.177	72	0.002		
alpha	Species	0.272	5	0.054	22.299	<0.001*
	Error (Species)	0.044	18	0.002		
	Time	0.121	1.479	0.082	8.734	0.003*
	Species * Time	0.119	7.397	0.016	1.718	0.144
	Error (Time)	0.249	26.628	0.009		
I_k_	Species	194592.651	5	38918.530	11.212	<0.001*
	Error (Species)	62480.808	18	3471.156		
	Time	86722.464	4	21680.616	5.549	0.001*
	Species * Time	378151.575	20	18907.579	4.839	<0.001*
	Error (Time)	281300.345	72	3906.949		
rETR_max_	Species	27906.053	5	5581.211	56.787	<0.001*
	Error (Species)	1769.104	18	98.284		
	Time	14831.341	4	3707.835	39.288	<0.001*
	Species * Time	21566.338	20	1078.317	11.426	<0.001*
	Error (Time)	6795.025	72	94.375		

* Significant difference.

## Data Availability

Data available from corresponding author upon request.
